# Improving activity recognition using a wearable barometric pressure sensor in mobility-impaired stroke patients

**DOI:** 10.1186/s12984-015-0060-2

**Published:** 2015-08-25

**Authors:** Fabien Massé, Roman R. Gonzenbach, Arash Arami, Anisoara Paraschiv-Ionescu, Andreas R. Luft, Kamiar Aminian

**Affiliations:** Laboratory of Movement Analysis and Measurement, Ecole Polytechnique Fédérale de Lausanne, Station 11, 1015 Lausanne, Switzerland; Department of Neurology, University Hospital of Zurich, Frauenklinikstrasse 26, 8091 Zürich, Switzerland

## Abstract

**Background:**

Stroke survivors often suffer from mobility deficits. Current clinical evaluation methods, including questionnaires and motor function tests, cannot provide an objective measure of the patients’ mobility in daily life. Physical activity performance in daily-life can be assessed using unobtrusive monitoring, for example with a single sensor module fixed on the trunk. Existing approaches based on inertial sensors have limited performance, particularly in detecting transitions between different activities and postures, due to the inherent inter-patient variability of kinematic patterns. To overcome these limitations, one possibility is to use additional information from a barometric pressure (BP) sensor.

**Methods:**

Our study aims at integrating BP and inertial sensor data into an activity classifier in order to improve the activity (*sitting*, *standing*, *walking*, *lying*) recognition and the corresponding body elevation (during climbing stairs or when taking an elevator). Taking into account the trunk elevation changes during postural transitions (sit-to-stand, stand-to-sit), we devised an *event-driven* activity classifier based on fuzzy-logic.

Data were acquired from 12 stroke patients with impaired mobility, using a trunk-worn inertial and BP sensor. Events*,* including walking and lying periods and potential postural transitions, were first extracted. These events were then fed into a double-stage hierarchical Fuzzy Inference System (H-FIS). The first stage processed the events to infer activities and the second stage improved activity recognition by applying behavioral constraints. Finally, the body elevation was estimated using a pattern-enhancing algorithm applied on BP. The patients were videotaped for reference. The performance of the algorithm was estimated using the Correct Classification Rate (CCR) and F-score. The BP-based classification approach was benchmarked against a previously-published fuzzy-logic classifier (FIS-IMU) and a conventional epoch-based classifier (EPOCH).

**Results:**

The algorithm performance for posture/activity detection, in terms of CCR was 90.4 %, with 3.3 % and 5.6 % improvements against FIS-IMU and EPOCH, respectively. The proposed classifier essentially benefits from a better recognition of *standing* activity (70.3 % versus 61.5 % [FIS-IMU] and 42.5 % [EPOCH]) with 98.2 % CCR for body elevation estimation.

**Conclusion:**

The monitoring and recognition of daily activities in mobility-impaired stoke patients can be significantly improved using a trunk-fixed sensor that integrates BP, inertial sensors, and an event-based activity classifier.

## Introduction

Stroke impacts approximately 17 million people worldwide every year [1]. Post-stroke survivors are mostly affected by mobility impairments, due to ataxia or hemiplegia, and consequences of lesion in the motor cortex following the stroke. Recovery of motor function requires intensive physical rehabilitation which must be tailored to the patient for better efficacy. Currently, therapeutic decisions are usually based on clinical assessment of motor function using functional tests such as the Berg Balance Scale (BBS) for balance assessment [2] or Timed Up and Go (TUG) for gait and balance evaluation [3], or on patient reports including QoL questionnaires such as the generic Stroke Impact Scale [4] or Stroke-specific Quality of Life [5]. Although useful and currently used in clinical practice, it is recognized that these evaluations may have some limitations. The QoL scores may be biased by the subjective interpretation of questions and the patient’s state-of-mind. The clinical functional tests are performed only in a hospital setting and may not reflect the actual motor performance of patients in everyday life. For example, a patient may show good balance and stable gait during the clinical exam, report good mobility in daily-life, however in reality he/she might avoid long distance walking or climbing stairs. Activity monitoring in everyday life is therefore expected to provide a more comprehensive assessment of physical functioning and QoL of post-stroke patients.

The unobtrusive monitoring of basic daily activities in the real-life environment has been extensively investigated over the last decade, along with the development and spread of wearable technologies. Daily activities were successfully monitored using a set of multiple inertial sensors (accelerometers and gyroscopes) placed at key body locations in patients with chronic pain [6], or stroke [7, 8]. Sensors placed on the trunk were used to detect lying and walking periods, and to characterize postural transfer such as *sit-to-stand* and *stand-to-sit* (STS) transitions [9], relevant for functional recovery assessment after stroke [10, 11]. Inertial sensors (accelerometers) on the thigh allowed distinguishing sitting from standing posture, while sensors on the shank/foot (gyroscopes) were used for detailed evaluation of the gait pattern. However, placing multiple sensors on the patient’s body may lead to discomfort and hence hinder their ability and willingness to perform normal daily activities. Given this limitation, a number of studies were dedicated to the development of activity monitors using a single sensor configuration [12–14]. Although the current activity monitors are accurate in recognizing dynamic activities (walking and running), their abilities to classify static postures (standing vs. sitting or sitting vs. lying) remain limited. For instance, an accelerometer placed on thigh cannot distinguish accurately sitting from lying [15, 16]. Furthermore, a trunk-located inertial sensor can distinguish various basic activities (lying, sitting, standing, and walking) but with limited performance, due to the variability of movement pattern across activities and patients [13, 17]. A possible solution to increase the performance of these algorithms is to use an additional sensor modality such as the barometric pressure (BP). BP provides an estimate of the sensor’s absolute altitude, which can be particularly useful for distinguishing transitions between activities involving altitude/body elevation changes (e.g. up/down level walking, stair claiming, STS transitions). This approach can result in detecting additional activities, for example the evaluation of patients mobility while climbing the stairs which is a relevant outcome for post-stroke recovery [18]. Lester et al. [19] and Moncada-Torres et al. [20] proposed activity recognition algorithms including BP-based stair climbing detection algorithm but the results were validated only on healthy controls.

In addition to the sensor configuration (number and placement), a methodological approach to recognize/classify the activities from the raw sensor data is crucial. The most common approach is the *epoch-based classification* [21], i.e. sensor data are split into fixed-length epochs, and based on extracted features machine learning techniques are applied to classify each epoch into an activity. Another approach is the *event-based classification*, which consists in *detection* and *classification* of key events such as postural transitions, start/end of walking and lying periods. Following this approach, Salarian et al. [22] incorporated postural transition-specific knowledge into a fuzzy logic based activity recognition algorithm as a way to improve the classification performances. This algorithm is based only on the information from a single inertial sensor fixed on the trunk therefore, the accuracy of STS classification is limited.

The present work is based on the following hypotheses: (1) changes in trunk elevation during STS postural transitions, inclined walking and stairs climbing can be detected by a multimodal sensor including inertial (accelerometers, gyroscopes) and BP sensing; (2) this information can be used to devise an improved event-based activity classification algorithm. We propose a wearable activity monitoring system based on single trunk-worn multimodal sensor system (Inertial Measurement Unit-IMU and BP) and a fuzzy logic based activity classifier that exploits fused information from the sensors. The classifier accounts for behavioral constraints and, in addition, estimates the body elevation (flat, up and down) during standing and locomotion.

## Method and materials

This section first describes the data collection protocol carried out on mobility-impaired stroke population. Then, the different steps of activity recognition algorithm, including event and transition detectors and a Hierarchical Fuzzy Inference System (H-FIS), are described. Finally, the assessment of algorithm performance and the validation procedure is specified.

### Data collection

The data were collected at the Kliniken Valens rehabilitation center (Valens, Switzerland) on 12 mobility-impaired stroke patients (7 females and 5 males/age = 59.6 ± 13.6 y.o./height = 170.1 ± 9.10 cm/weight = 73.9 ± 14.1 kg) suffering from hemiplegia due to an ischemic or a hemorrhagic stroke. Eight out of twelve patients were able to walk independently but four needed assistance (cane or a walking frame).

Each patient was equipped with a set of wearable sensors and performed daily-life activities as instructed by the physician, for approximately 30 min (33.4 ± 9.4 min), depending on the patient’s fitness condition. The objective was to include a set of basic activities of daily living: short and long walking episodes, walking up and down the stairs, taking the elevator, postural changes between lying, standing and sitting with and without arm movements. Various seats were included in the activity path: arm chair, bed side, sofa, armless chair, and stool. The set of daily-life activities included walking along a corridor, watching TV, washing hands, eating, pouring and drinking water, sleeping, shoe lacing, reading the newspaper, and putting jacket on and off. These activities were performed in a semi-structured protocol to better correspond to real-life situation [23]. In other words, the activities were suggested in such a way that flexibility was given on when and how to be performed. For instance, “watching TV” required the patient to walk towards the TV area, sit down on the sofa, use the remote control for turning on the TV and relax while watching TV. Furthermore, the number and order of the instructed activities were not scripted in advance. During the trial, each patient was videotaped for algorithm validation purpose. The study was approved by the ethical committee “Ethikkommission des Kantons St. Gallen” (St Gallen, Swiss Canton, Switzerland).

### Measurement system and validation reference

The measurement system consisted of a small wearable sensor module (Physilog® 10D Silver, GaitUP, CH) attached to the patient’s trunk (sternum) using hypoallergenic breathable band (Opsite FlexiFix). The device recorded to an on-board memory card the signals from an inertial sensor (3D accelerometer and 3D gyroscope) at 200Hz, and from a BP sensor at 25 Hz. The precision of the BP sensor is 1.2 Pa (~10 cm) according to the manufacturer datasheet [24]. The signals from sensors were first resampled at the same frequency of 40Hz to allow for faster processing. This frequency is sufficiently high to extract activity features [12, 25]. Moreover, the wearable sensors were aligned with the body segments by a functional calibration procedure based on two defined postures: lying down on a bed and standing upright against a wall. First, the orientation of the gravity vector in the sensor frame at these two specific postures was recorded. The rotation matrix which maps the corresponding frame axes of accelerometer sensor to these vectors was then obtained. This procedure enabled to virtually align the sensor frame with the body frame, in order to ensure robustness against sensor misalignment across patients [26].

### Activity recognition

**Fig. 1:**
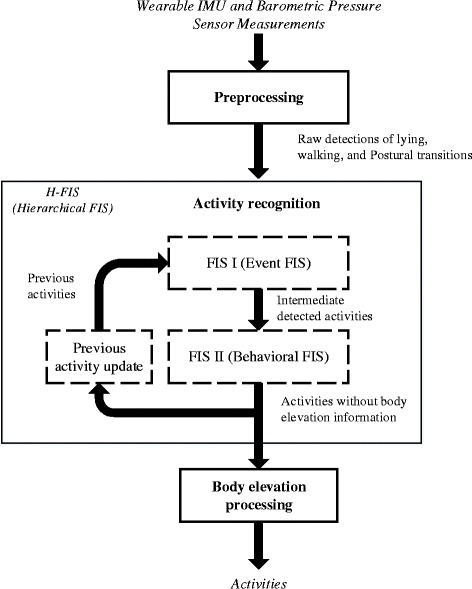
Block diagram of the activity recognition algorithm. Following the acquisition of the IMU and barometric pressure signals from the wearable device, the acquired signals are then preprocessed to extract key events (postural transitions, steps, lying periods). Then these events are combined into a hierarchical FIS to output the basic activities. The output of FIS II, i.e. the detected activities were fed into the decision tree for body elevation estimation and fed back into the FIS I for the detection of next activities

Unlike *epoch-based classifiers*, the proposed *event-driven activity classifier* relied on preprocessed events such as the start/end of walking and lying periods and STS postural transitions. After detection, these events were processed through a two-stage H-FIS to classify the basic daily-life posture/activities: *lying*, *sitting*, *standing*, and *walking*. While the first stage (FIS I - Event FIS) was in charge of translating the detected events into activities, the second stage (FIS II - Behavior FIS) was designed to apply linguistic behavioral constraints for improving the recognition of activities as inferred by the first stage. The *standing* and *walking* activities were further categorized by a decision tree according to the estimated elevation level: flat *level standing*, *elevator down* (standing with a downward elevation change), *elevator up* (standing with an upward elevation change), flat *level walking*, *walking downstairs*, and *walking upstairs.* A schematic of the algorithm is illustrated in Fig. 1.

#### Preprocessing: Event detection and characterization

##### Detect lying and walking periods

The start/end of lying periods were identified using the trunk angle with respect to gravity, estimated from the accelerometer. The *start* was defined when the trunk angle drops below a defined threshold θ_Lying_ = 45° for more than 10 s, and the *end* when the trunk angle goes above θ_Lying_ for more than 10 s.

The start/end of walking periods were detected according to the algorithm devised by Najafi et al. [27]. The norm of the trunk acceleration signal was band-passed from 1 to 5Hz using a second-order Butterworth filter. From this signal, all the peaks located above the threshold ∆â_walking_ were selected and considered as potential heel strike events if duration between them was at least ∆T_walking_steps_ = 0.25 s. Then, consecutive heel strikes within the time interval of ∆T_walking_group_ = 3 s were grouped to form a walking period. This condition was defined according to the definition of walking activity as at least three consecutive steps.

##### STS transitions detection and classification

The STS postural transition detection and characterization relied on an algorithm [28] estimating two probabilities for each candidate transitions: 1) the probability of a candidate transition (P_Tr_) to be true; and 2) the probability of a candidate transition type (P_Type_) to be a sit-to-stand transition. These probabilities were estimated using the logistic regression. Further, the features characterizing the transitions were used in the next stage to help recognizing the activity type.

#### Two-stage Hierarchical Fuzzy Interference System (H-FIS)

A fuzzy inference system is generally defined by a set of membership functions to transfer its inputs into fuzzy (linguistic) variables, a set of “If-Then” rules to fuse the fuzzy variables and map the antecedents to consequences, an implication and aggregation operator, and finally a de-fuzzification method (for more detail please see Appendix). The H-FIS was initially designed for the control of complex systems [29] and consisted in a cascade of several FISs for which the most influential system variables are used by the first level, the next most influential variables at the second level and so on [30]. This cascade of FISs was meant to drastically reduce the number of rules in the system.

The presented H-FIS was composed of two stages as described in Fig. 1. The Event stage (Event-FIS) handles the translation from events to activity and the Behavioral stage accounts for biomechanical constraints to improve the recognition of activities classified in the first stage. They were both implemented as Mamdani-type FISs [31]. Definitions of the inputs and outputs of the FISs are listed in Table 1.

**Table 1:** Definition of inputs and outputs of the H-FIS

Name	Description
Inputs	
PrevAct	Previous activity: the activity preceding the activity being evaluated by the algorithm
CurrAct	Current activity: the activity being evaluated by the algorithm
NextAct	Next activity: the activity following the activity being evaluated by the algorithm
Transition	Transition detection probability: Computed through a logistic regression model, it provides a continuous value (P_Tr_) from 0 to 1 representing the probability for a transition to be respectively “Not Detected” and “Detected”
Transition type	Transition type probability: Computed through a logistic regression model, it provides a continuous value (P_Type_) from 0 to 1 representing the probability for a transition to be respectively of a type “Sit-to-stand” and “Stand-to-Sit”
PrevDur	Duration of the previous activity being processed
CurDur	Duration of the current activity being processed
NextDur	Duration of the activity following the activity being processed
AltitudeChange	Altitude change corresponds to the change in elevation around the transition time. It is computed on the barometric pressure signal through transition feature extraction algorithm [28]
AltitudeIQR	Altitude Inter-Quartile Range is computed over the duration of the activity on the altitude signal (derived from the barometric pressure signal)
Outputs	
Event activity	Event activity represents the output of FIS I (this is the current activity recognized in this stage of classification)
Behaviour activity	Behavior Activity represents the output of FIS II and thus the final output of the H-FIS (this is the current activity recognized at the end of second stage of classification)

#### Event FIS

The set of information used as inputs were: the previous activity (*PrevAct*), the current activity (*CurrAct)*, the postural transition detection probability P_Tr_*(Transition)*, the postural transition classification probability P_Type_ (*TransitionType)*, and the altitude difference before and after transitions, more specifically the difference between the averaged values during the 10 s before and after transition time (*AltitudeChange*). The output of this stage was then fed as an input into the Behavior FIS.

**Fig. 2:**
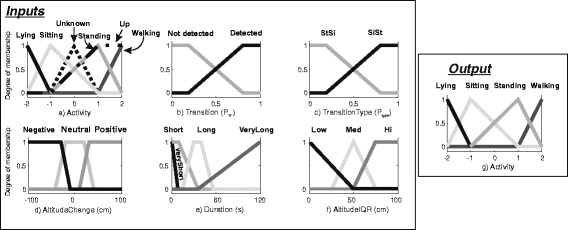
Definition of membership functions for the first and second stages. For plot **a**) through **f**), the horizontal axis represents the input value (for plots **a**) through **f**) whereas, for plot **g**), it represents the output value. The vertical axis denotes the degree of membership for each of the inputs/output

The membership functions for the different states of the fuzzy variables are described in Fig. 2. Six membership functions were defined for the fuzzy variables *PrevAct* and *CurrAct* depending upon the considered activities (Fig. 2a): *lying*, *sitting*, *standing*, *walking, up* and *unknown*. Two membership functions were defined for both *Transition* (Fig. 2b) and *TransitionType* (Fig. 2c) similar to [22]. Three trapezoidal membership functions (Fig. 2d) were defined for the input *AltitudeChange*, designed with slopes accounting for the precision of the barometric pressure sensor. Prior to the processing of the inputs in the FIS, the *CurrAct* was initialized as *lying* or *walking* if a lying or walking period was detected at the preprocessing stage, or as *unknown* otherwise.

Furthermore, rules for the lying-to-sitting and lying-to-standing were added as the logistic regression-based transition models did not account for these transition types. Not all possible combinations of fuzzified inputs were used as antecedents to build the rule base since firstly, some of them are mutually exclusive due to the biomechanical constraints. For instance the antecedent of if the previous activity is lying and current activity is walking was excluded since a direct transition from sitting to walking is impossible unless through siting and standing events. Secondly, knowing the strengths and weaknesses of IMUs from previous studies [17, 22] that indicated the difficulty in detection of transitions comparing to walking and lying events, more emphasis was given to a subset of antecedents to improve this aspect (more than half of the rules).

The fuzzy rules are presented in Table 2. The steps for obtaining the output are illustrated in Appendix. A feedback loop was implemented to update the *PrevAct* using the output of the Behavior FIS.

**Table 2:** Fuzzy rules for the event FIS

Input					Output
PrevAct	CurrAct	Transition	Transition type	Altitude change	Event activity
	Lying				Lying
	Walking				Walking
Lying	Unknown			Positive	Standing
Lying	Unknown			Not Positive	Sitting
Sitting	Unknown	Detected	SiSt		Standing
Sitting	Unknown	Detected	StSi		Sitting
Sitting	Unknown	Not Detected			Sitting
Up	Unknown	Detected	StSi		Sitting
Up	Unknown	Detected	SiSt		Standing
Up	Unknown	Not Detected			Standing

#### Behavior FIS

The second stage applied behavior-inspired constraints to the output of the first stage in order to improve the overall classification performance. The following behavioral constraints were considered while building the FIS rules set:

- It is likely that the activity detected in the Event-FIS is the true activity.
- It is unlikely to have a very short walking period (∆T_walk_) preceded and followed by a long period of siting. This would be probably moving during siting.
- It is unlikely that a person walks for a very short time after lying or sitting, especially if there is no change of altitude.
- It is likely that if the detected activity is sitting for a relatively short time and there is a high change of altitude during the activity, the activity is standing (going up/down the elevator). This time constraint was to avoid known long-term variations of barometric pressure.
- It is likely that if a person is standing (and not moving at all) for a lengthy duration (∆T_standing_), the activity is actually sitting [32].

These behavioral constraints were applied to a set of fuzzy variables and rules, as displayed in Table 3. Similar to Event FIS, not all the antecedents were used to build up the rule base but the biomechanically meaningful rules were hand engineered. These rules can transfer the result of the Event FIS (the recognized current activity) to its output (if and only if its first four rules – labelled as a) in Table 3 – were activated) or modulate the Event FIS output (through the activation of its next set of rules). The following inputs were added: *NextAct* that accounts for the next activity as computed by the previous stage and shares the same membership functions as *CurrAct. PrevDur*, *CurrDur*, and *NextDur* that corresponds to the duration of the previous, current and next activity, respectively. Four membership functions were also defined to account for different types of activities (Fig. 2e): *VeryShort* (0 s to ∆T_walk_ = 7 s) for spurious activities than may need to be filtered out, *Short* for slightly longer activities (0 s to 30s), *Long* (15 s to 60s) and *VeryLong* (30s to ∆T_standing_ = 120 s) for resting activities. An additional input was *AltitudeChange* corresponding to the change in altitude around the transition as defined for the Event-FIS. Four membership functions were associated with *AltitudeChange*: Negative, Neutral for handling spurious walking activities, and Positive. Furthermore, *AltitudeIQR*, the inter-quartile range (IQR), was computed from the altitude signal during the activity and added as input. Three membership functions were associated to the *AltitudeIQR* (Fig. 2f): *Low*, *Med*, and *Hi* depending on the assumed elevation change during activities. This latter is used for correcting the possible misclassification of sitting as standing when taking the elevator for instance. These fuzzy variables were essentially introduced to prevent Elevator (*standing*) activities to be misclassified as *sitting*. The association between the constraints and the rules are also indicated in the Table 3. Note that a “NOT” keyword preceding the membership function means that the complementary (one minus the original) membership function is used.

**Table 3:** Fuzzy rules for the behaviour FIS

Rule	Input								Weight	Output
	Prev act	Curr act	Next act	Prev dur	Cur dur	Next dur	Alt change	Altitude IQR		Event activity
a)		Lying							0.5	Lying
a)		Walking							0.5	Walk
a)		Sitting							0.5	Sitting
a)		Standing							0.5	Standing
b)	Sitting		Sitting	Not Short	Not Short	Not Short			0.5	Sitting
c)	Sitting	Walking					Not Positive		0.75	Sitting
c)	Lying	Walking					Not Positive		0.75	Lying
d)		Sitting			Not Very Long			Very Positive	0.75	Standing
e)		Standing			Very Long				1	Sitting

The letters in the first column indicate the association between rule and the constraint, as listed in this section

Furthermore, weights, defined based on expert knowledge, were associated with the rules to change their contributions according to the confidence level of the rule in order to favor specific rules against others. For this stage the mean of maximum is used to defuzzify the rule outputs. This method was selected instead of the centroid method as used in the first stage to favor the rule contributing the most (highest output value). The crisp class of the *Activity* was computed after the defuzzification stage according to Table 4. The effect of weights on the output is further detailed in the Appendix.

**Table 4:** Output of the H-FIS: the crispation of a defuzzified output translates a value to a class

Defuzzified output value	Activity class (crisp value)
[−2; 1.5)	Lying
[−1.5; 0)	Sitting
[0; 1.5)	Standing
[1.5; 2]	Walking

#### Body elevation classification and altitude fitting

An activity may contain a subset of body elevation. For instance, walking includes level walking, and non-level walking such as climbing up the stairs. The BP sensor is sensitive to body elevation, however, the patient’s slow dynamics during stair climbing combined with the low signal-to-noise ratio and the influences of external perturbations of the BP sensor impede the recognition of elevation without appropriate pattern enhancing techniques such as signal pattern fitting. The third stage of the algorithm includes therefore a decision tree combined with a sinus-fitting algorithm built to detect accurately the body elevation and distinguish level walking from going upstairs/downstairs for walking activity; and stand still from elevator up/down for standing activity.

**Fig. 3:**
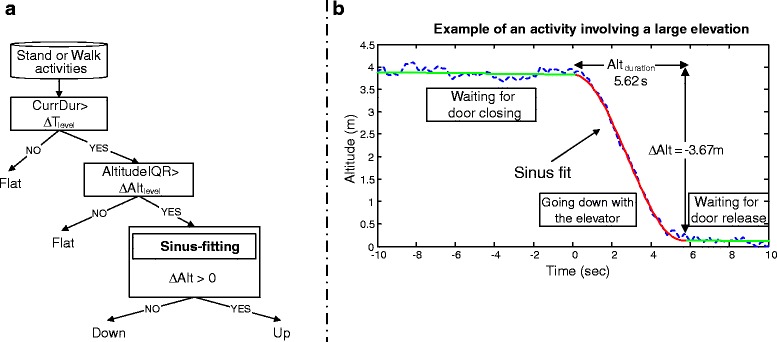
Classification of body elevation. **a** Decision tree for the classification of body elevation / **b** Example of an activity involving a large elevation (Elevator Down)

The BP signal was first converted to altitude (*Alt*) using the barometric formula [33] then the pattern of elevation was enhanced using a sinusoidal fitting model similar to model used in STS detection [28]. The sinus fitting function (*S*_*Alt*_) was modeled as follows:

1 $$ \begin{array}{c}\hfill {S}_{Alt}(t)={\Delta}_{Alt}*E\left(\frac{t-Al{t}_{delay}}{Al{t}_{duration}}\right)+Al{t}_{drift}*t+Al{t}_{offset}\ \hfill \\ {}\hfill with\ E(t)\left\{\begin{array}{ll}-1/2\hfill & if\ t\le -1/2\hfill \\ {}1/2* \sin \left(\pi t\right),\hfill & if-1/2<t\le 1/2\hfill \\ {}+1/2\hfill & t>1/2\hfill \end{array}\right.\hfill \end{array} $$

where the model parameters ∆_*Alt*_, *Alt*_*duration*_*,*, *Alt*_*offset,*_*Alt*_drift_, *Alt*_delay_ are depicted in Fig. 3. They represent over the course of the activity the change in altitude, the duration of the part of the activity that involves a potential elevation change, the potential elevation drift and the elevation offset, respectively. For each activity, the model was obtained from the altitude data (over the duration of the activity being processed) using the “Trust-region reflective” optimization procedure [34].

The parameters ∆_*Alt*_, *Alt*_*duration*_*,* and *Alt*_*offset*_ were optimized in order to smoothen the signal. The parameter *Alt*_drift_, was bounded during the optimization with limits calculated from the datasheet of the BP sensor (MS5611-BA01, Measurement Specialties). This also allows accounting for slow variations of barometric pressure due to weather changes. Note that ∆_*Alt*_ could take positive or negative values depending on whether the signal was shaped as a rising edge or falling edge respectively. A decision tree based algorithm was designed to further classify the *standing* and walking *activities* according to the estimated altitude (Fig. 3a). First, these activities were classified as *flat* or *up/down* using the information from two variables, the altitude change (*AltitudeIQR*) and the duration of the new altitude level (*CurrDur*). Then, the up/down class was classified as *up* or *down* according to the sign of *∆*_*Alt*_ and the value of parameter *Alt*_*duration..*_ Fig. 3b illustrates an example of altitude fitting corresponding to *elevator down* activity.

### Benchmarked algorithms

#### Epoch-based algorithm

The epoch-based algorithm (EPOCH) was inspired by a recent algorithm [20] which processed the data after splitting into N_epoch_ epochs of ∆T_epochs_ (5 s) and classified activities based on features extracted from each epoch. This algorithm was selected as it also proposed to classify activities using barometric pressure and inertial sensors and it was validated on an elderly patient population. The feature set consisted of 120 features including frequency, amplitude and temporal features derived from the inertial sensors. To avoid over-fitting, the feature set was reduced using ReliefF algorithm [35] to K features to form the minimal feature set Ω_epochs_ = {N_epoch_ × K features}. These features were then fed into a machine learning classifier (Classification tree) [36]. Following the leave-one patient-out cross validation procedure (described in the validation section) each epoch was finally classified as either *walking*, *lying*, *sitting* or *standing*.

#### Fuzzy-based algorithms

The FIS described in Salarian et al. [22], called here FIS-IMU, was essentially designed to compensate for classification errors in the recognition of postural transitions. It uses a subset of the previously described fuzzy variables and membership functions. Although it uses the same events (start/end of walking/lying and STS transitions), the logistic regressions used for computing the probabilities P_Tr_^inertial^, P_Type_^inertial^ relied only on information from inertial sensors. The classifier FIS-IMU did not account for altitude features in the computation of the probabilities P_Tr_^inertial^ and P_Type_^inertial^. To fairly estimate the added value of BP sensor, we used the probabilities P_Tr_ and P_Type_ instead of P_Tr_^inertial^ and P_Type_^inertial^, in an augmented classifier FIS-IMUBP, to account for the effect of classification improvement thanks to the altitude features.

### Validation

#### Strategy and procedure

##### Activity classification

The performances of five algorithms were evaluated and compared: H-FIS (Event FIS + Behavior FIS), Event FIS, and state-of-the-art FIS-IMU as described by Salarian et al. and its BP-augmented version FIS-IMUBP, and EPOCH, a traditional epoch-based modeling approach (see Table 5). Furthermore, the output of the Event FIS was also computed separately from the H-FIS to estimate the performance improvement by the second stage.

**Table 5:** Classifier validation procedure for activity recognition: summary table

#	Classifier	Acronym	Sensors	Validation
1	Event + Behavior FIS	H-FIS	Inertial and barometric	Full dataset
2	FIS Salarian et al.	FIS-IMUBP	Inertial and barometric	Full dataset
3	Event FIS	Event-FIS	Inertial and barometric	Full dataset
4	FIS Salarian et al.	FIS-IMU	Inertial	Full dataset
5	Epoch-based model	EPOCH	Inertial	Cross validation

##### Body elevation

With regard to the body elevation classifier, the performance improvement by using a sinus-fitting algorithm in an event-based activity recognition algorithm was evaluated using the following comparison strategies:

- H-FIS: the activity output of the H-FIS was combined with a decision tree classifier as displayed in Fig. 3. Furthermore, in case an elevated activity was detected, the portion located inside the sinus fit (red line in Fig. 3) was labelled as *Up* or *Down* depending of the sign of ∆*Alt*. Its corresponding activity duration was consequently set to *Alt*_*duration*_. The remaining portion(s) of the activity (green line in Fig. 3) was considered as *Flat*.
- H-FISnoFit: the activity output of the H-FIS combined with the decision tree classifier. The decision was made using a single criterion: whether the maximum value of the altitude signal is reached before or after the minimum value of the signal over the duration of the activity.
- EPOCH-BP algorithm [20]: where EPOCH feature set was augmented with altitude features, namely IQR, standard deviation, slope, and range of the altitude signal during the epoch [20].

Each patient was videotaped during the trial with a camera synchronized with the wearable system. The video recordings were annotated to form the reference activity set, Ω_reference_.

All FIS-based activity classifiers were validated against the full dataset (no training/testing dataset split) as no parameter was required to be learnt from data to build the FIS in our implementations. The EPOCH classifiers were cross validated using a similar leave-one-out cross validation methodology as presented before.

#### Validation metrics

From the validation procedure described before, a confusion matrix was estimated for each classifier. Various metrics were extracted from these confusion matrices including: True Positive Rate also called Recall or Sensitivity (SEN), True Negative Rate also called Specificity (SPE), Positive Predictive Value (PPV) also called Precision, Negative Predictive Value (NPV), and the Correct Classification Rate (CCR) [37]. The F-Score, defined as the harmonic mean between the sensitivity and the positive predictive value, was used for overall performance evaluation [37]:

$$ {F}_{score}=2\times \frac{SEN \bullet PPV}{SEN+PPV} $$

The performances of the classifiers were compared in two conditions. First, for each classifier the confusion matrices across all datasets (N_patients_ = 12) were aggregated to calculate the corresponding *CCR* and F-score; these overall performance scores were comparatively evaluated between the classifiers. Second, the performance scores were evaluated for each dataset/patient.

Non-parametric multiple comparison (Friedman’s test [38]) was conducted to test for the statistical significance of difference of the performance scores [39] estimated with the different classifiers (each time for the same sample of 12 datasets). The level for statistical significance was set to p < 0.05.

## Results

### Overall performance

#### Activity recognition

The confusion matrices are presented in Table 6 along with the validation metrics. The H-FIS outperformed the FIS-IMU by 3.3 %, the FIS-IMUBP by 1.0 %, and the EPOCH by 5.6 %. This is mostly due to an improvement of the F-score (from 2.3 % for FIS-IMUBP up to 28.0 % for EPOCH) for the *standing* activity, consecutive to an improvement of PPV (+3.5 %) with respect to FIS-IMUBP and a 45.1 % drop of sensitivity for the EPOCH (80.6 % for H-FIS vs. 35.5 % for EPOCH). Furthermore, the effect of adding behavior constraints improved the overall accuracy of H-FIS vs. Event-FIS by 8.5 % essentially by providing a better distinction between sitting and standing posture.

**Table 6:** Confusion matrices for the recognition of the activities along with the corresponding validation metrics for the five classifiers expressed in percent

		Classification									
		Lying	Sitting	Standing	Walking	SEN	SPE	PPV	NPV	F-score	CCR
		H-FIS									
Reference	Lying	1022	37	2.8	12.2	95.2	99.7	94.5	99.8 %	94.8 (96.9 ± 5.0)	90.4 (91.4 ± 6.6)
	Sitting	37.3	11975	737.1	286.1	91.9	95.5	96.0	90.8 %	93.9 (95.7 ± 8.4)	
	Standing	22.2	245.3	2066	228.9	80.6	94.3	62.7	97.6 %	70.5 (71.4 ± 11.3)	
	Walking	0	211	490.9	6667.5	90.5	96.8	92.7	95.8 %	91.6 (91.0 ± 6.0)	
		FIS-IMUBP									
Reference	Lying	1022	21.1	17.1	13.8	95.2	99.7	94.5	99.8 %	94.8 (96.9 ± 5.0)	89.4 (89.8 ± 5.9)
	Sitting	37.3	11729.9	940.9	327.4	90.0	95.4	95.9	88.9 %	92.8 (93.6 ± 7.0)	
	Standing	22.2	249.8	2060.6	229.8	80.4	93.4	59.2	97.6 %	68.2 (68.2 ± 14.7)	
	Walking	0	230.1	461.7	6677.6	90.6	96.6	92.1	95.9 %	91.4 (91.0 ± 6.0)	
		Event-FIS									
Reference	Lying	1022	33.4	4.8	13.8	95.2	99.7	94.5	99.8 %	94.8 (96.9 ± 5.0)	81.9 (84.4 ± 12.0)
	Sitting	37.3	9829.6	2841.2	327.4	75.4	96.9	96.7	76.9 %	84.7 (86.4 ± 29.5)	
	Standing	22.2	139.3	2171.1	229.8	84.7	84.3	39.2	97.9 %	53.6 (65.8 ± 36.7)	
	Walking	0	167.9	523.9	6677.6	90.6	96.6	92.1	95.9 %	91.4 (91.0 ± 6.0)	
		FIS-IMU									
Reference	Lying	1022	6.1	32.1	13.8	95.2	99.7	94.5	99.8 %	94.8 (96.9 ± 5.0)	87.1 (86.8 ± 6.2)
	Sitting	37.3	11231.1	1439.7	327.4	86.2	95.4	95.7	85.3 %	90.7 (90.2 ± 8.0)	
	Standing	22.2	301.7	2008.7	229.8	78.4	90.9	50.6	97.2 %	61.5 (62.4 ± 23.0)	
	Walking	0	201.9	489.9	6677.6	90.6	96.6	92.1	95.9 %	91.4 (91.0 ± 6.0)	
		EPOCH									
Reference	Lying	892	120	28	24	83.8	99.3	84.5	99.3 %	84.2 (90.7 ± 12.4)	84.8 (84.0 ± 5.4)
	Sitting	124	11980	608	484	90.8	83.1	86.5	88.3 %	88.6 (88.3 ± 10.6)	
	Standing	40	1264	908	348	35.5	96.3	53.0	92.7 %	42.5 (39.6 ± 15.9)	
	Walking	0	484	168	6752	91.2	94.9	88.7	96.1 %	90.0 (91.3 ± 6.0)	

Each confusion matrix is expressed in seconds

For the CCR and the F-score, the median and interquartile range are provided (computed across patients)

*SEN* Sensitivity, *SPE* Specificity, *PPV* Positive Predictive Value, *NPV* Negative Predictive Value, *CCR* Correct Classification Rate

#### Body elevation

The activity confusion matrices and validation metrics are presented in the Table 7 for comparison between the EPOCS-BP, the H-FIS, and H-FISnoFIT. The H-FIS performed better in terms of overall accuracy (98.2 %) essentially due to a high F-score which reached 72.6 % on average for the four activity levels. The average F-score was 64.5 % for the H-FISnoFIT approach and only 50.8 % for the EPOCH-BP approach.

**Table 7:** Confusion matrices after the classification of the activity levels along with the corresponding evaluation metrics

		Classification						
		Flat	Elevator down	Elevator up	Stairs down	Stairs up	F-score	CCR
		H-FIS						
Reference	Flat	23093.8	39.3	31	111.7	70.1	99.0 (99.0 ± 1.3)	98.2 (98.0 ± 1.5)
	Elevator Down	40.8	108.6	13.1	0	0	70.0 (83.7 ± 10.5)	
	Elevator Up	79.5	0	190.4	0	0	75.5 (82.9 ± 12.1)	
	Stairs Down	33	0	0	166.3	0	69.7 (78.4 ± 33.3)	
	Stairs Up	52.8	0	0	0	188	75.4 (78.3 ± 15.2)	
		EPOCH-BP						
Reference	Flat	23144	68	40	140	152	98.8 (98.9 ± 0.1)	96.9 (97.2 ± 0.2)
	Elevator Down	32	88	52	0	0	45.8 (66.7 ± 54.1)	
	Elevator Up	32	56	76	0	0	45.8 (67.6 ± 27.7)	
	Stairs Down	48	0	0	124	4	56.2 (57.1 ± 31.3)	
	Stairs Up	48	0	0	0	124	55.3 (61.5 ± 15.8)	
		H-FISnoFIT						
Reference	Flat	22682.6	18.7	25.7	57.6	37.7	98.4 (98.5 ± 1.7)	96.8 (96.5 ± 2.1)
	Elevator Down	188.5	129.2	13.8	0	0	53.9 (71.3 ± 27.0)	
	Elevator Up	189.6	0	195	0	0	63.0 (72.4 ± 29.0)	
	Stairs Down	119	0	0	208.6	0	68.9 (60.7 ± 11.9)	
	Stairs Up	120.2	0	0	11.8	220.4	72.2 (73.6 ± 13.8)	

*Walking* and *standing* activities are separated in the confusion matrix to further characterize the error. Each confusion matrix is expressed in seconds. For the CCR and the F-score, the median and interquartile range are provided (computed across patients)

#### Statistical analysis

The improvement of the overall performance score (F-score) is also emphasized in Table 7, that presents the median and the inter-quartile range of F-scores and CCR over the 12 datasets.

When analyzing the statistical significance of differences of CCR metric between the compared classifiers, a significant difference (*p* < 0.05) was found between H-FIS and the models not featuring barometric pressure (FIS-IMU: *p* = 0.02, and EPOCH: *p* = 0.004). No significant difference was found between H-FIS and the other models (FIS-IMUBP: *p* = 0.24, and Event-FIS: *p* = 0.06). However, the H-FIS contributed in improving the CCR for most of the patients: CCR improvements were observed in 8 out of 12 patients for the FIS-IMUBP and 6 out of 12 patients for the Event FIS. For this latter, the CCR remained unchanged for 5 patients and decreased for the remaining one.

Furthermore, no significant difference was found between FIS-IMUBP and FIS-IMU (*p* = 0.24) and between Event-FIS, FIS-IMU and EPOCH (p = 0.56). Similar significance values were observed for the F-score of the *sitting* and *standing* activities, except that the F-Score of H-FIS approach had greater significance (p = 0.03) with respect to the FIS-IMU. For *lying*, no significant difference was observed across all the models. With respect to *walking*, the H-FIS’s F-score was significantly different (*p* = 0.01) with respect to the other classifiers.

## Discussion

This study presents a new activity recognition algorithm able not only to recognize the basic daily-life activities (*lying*, *sitting*, *standing*, *walking*) but also to distinguish the body elevation using barometric pressure: *Up* and *Down* the elevator for *standing* and *Up* and *Down* the stairs for *walking*. The recognition of daily activity was carried out by a double-stage hierarchical fuzzy logic inference system. While the first stage processed the events such as the start/end of lying or walking periods, and detected postural transitions, the second stage improved the activity recognition by providing a simple way to integrate the typical behavior of the subject and biomechanical constraints. Five algorithms were benchmarked on a dataset containing daily-living activities from 12 patients suffering from post-stroke mobility impairments. The validation was performed using the conventional classification metrics, i.e., SEN, SPE, PPV and F-score estimated for each activity and overall for the ensemble of activities.

The results presented in this study demonstrate the efficiency of the event-driven algorithms featuring the BP sensor. This is essentially because the event-driven architecture of H-FIS and the FIS-IMUBP enables to leverage the full potential of the barometric pressure at the postural transition time, i.e. the body altitude change. Furthermore, the H-FIS results were statistically compared with other methods across patient-specific dataset. A statistical significant difference (*p* < 0.05) was always found between H-FIS approach and the inertial-based approaches, highlighting an improvement in the recognition across all patients (N_patients_ = 12). Even if the difference between H-FIS and the evaluated state-of-the-art algorithms (FIS-IMU and EPOCH) in terms of overall CCR may appear minor, it results in a superior performance in classification of *standing* activity (minimum 9.0 % increase between H-FIS and these algorithms). Better performance for standing classification (distinction from sitting) was one of main objectives of the present study, since this information is important for the clinical assessment of patients’ recovery (standing is a ‘dynamic’ activity related to physical capacity [40]).

The dataset used in this study was composed of daily activities performed at the clinic in naturalistic conditions. Ganea et al. [13] highlighted a lowering of recognition performance when algorithms were applied to data collected in “real” daily-life context, mostly due to a decreased ability in recognizing STS transitions. The addition of the BP sensor in the present study allowed to overcome this limitation. The pressure-based STS recognition is less prone to pathology related changes of trunk movement patterns.

The ability of negotiating stairs is an important component in stroke patients’ physical recovery process [41]. The body elevation was therefore computed to distinguish different ambulatory strategies: taking the stairs or the elevator as opposed to (flat) *level walking* or *standing*. The CCR of the three considered approaches (H-FIS, H-FISnoFIT, EPOCH) was superior to 96.8 % for all algorithms, due to the high F-Score (>98.4 %) in the *Flat* class where most of the instances were located. This class unbalance characterized by more sample data for the *Flat* class yielded to very high CCR, despite moderate classification performance in the other class. Nonetheless, the H-FIS outperformed the benchmarked algorithms in terms of F-Score for all the other classes. The difference between the H-FIS and the H-FISnoFIT in terms of CCR can essentially be explained by the improvements in F-score over the *non-level* activity detection (72.6 % for H-FIS vs., 64.5 % for H-FISnoFIT). This was essentially due to the narrowing of the *non-level* activity duration using the sinus fitting functions which can be observed by the increase in PPV (66.8 % for H-FIS vs. 38.5 % for H-FISnoFIT). The exact time of elevator start-off movement was difficult to track using the video recording and may explain the few seconds wrongly-classified as *Flat* for the *Elevator Up/Down* activities.

Similarly, due to slow dynamics of trunk movement, the annotation around an activity transition was difficult and this may have worsened the results. Furthermore, each period containing more than three consecutive steps were annotated as walking. However, the walking detection algorithm was initially developed for healthy/fit elderly subjects [27] without mobility impairments. When applied for mobility impaired stroke patients, the algorithm might consider a slow walking period as *standing* (F-Score of 70 % for H-FIS). These factors may have adverse effects on results. Nonetheless, an F-score greater than 90 % was obtained for *walking*. Another limitation, occurring during the slow motion period within walking, is the lack of sensitivity at recognizing level walking from stair climbing. This was essentially because a patient climbing the stairs might stall for few seconds, which would then end the current walking session and start a new standing session followed by a walking episode. These periods may not reach the required amplitude threshold ∆Alt_level_ and hence not be classified as climbing activity. A solution could be either to have different thresholds according to the climbing activities or to group a sequence of consecutive *standing* and *walking* activities.

The benefit of applying behavior-inspired constraints was observed by comparing the H-FIS with the Event-FIS in terms of CCR (90.4 % for H-FIS vs 81.9 % for Event-FIS). This difference greatly lies in the rule related to the correction of very-long (∆T_standing_ = 2 min) standing postures (rule e). The removal of the corresponding rule (rule e) from the fuzzy rule set (listed in Table 3) led to a 7.2 % decrease of the H-FIS’ CCR. A similar constraint was applied by Salarian et al. (FIS-IMU) [22] to improve the recognition. This threshold (∆T_standing_) can be fine-tuned according to different pathologies based on the analysis of behavioral data collected in free-living environment [42]. Furthermore, the behavioral rule (rule d) enables only a short sitting activity to be considered as standing if a large and sudden change of altitude is detected. This timespan limit prevents specific actions such as sitting in a car moving on a mountain road to be wrongly classified as *standing*. It also blocks any interference stemming from daily-changes in atmospheric pressure due to their very low dynamics.

In this study, we selected one epoch-based machine learning algorithm (decision tree) which was more descriptive due to the use of decision tree. However, we tested as well various machine learning algorithms using Weka software [43] on the same feature-reduced dataset and with the same leave-one-patient-out cross-validation procedure applied. They all resulted in an overall performance for activity recognition similar to EPOCH, i.e. CCR smaller than 87.1 % (Decision Table: 82.5 % CCR; Naïve Bayes: 81.6 %; Random Forest, #Trees = 10: 87.1 %; K-Nearest-Neighbors, K = 10: 85.6 %) confirming the advantage of the event-driven algorithmic approach.

The goal of this study was not to optimize the fuzzy rules and operators for H-FIS classifier, but to introduce a methodological approach that incorporates BP sensors alongside with the inertial measurement as a way to improve the activity classification. The fuzzy rules were therefore hand-engineered in this investigation. However a global optimization algorithm or any hybrid-Fuzzy system with adaptation can replace each of the fuzzy blocks to improve the performance. Furthermore, fusing the epoch-based algorithm with the H-FIS could also improve the performance of the presented system. For instance, for a prolonged activity, an epoch-based algorithm could split this activity into multiple epochs and then infer the activity by combining the results across the epochs using a meta-classifier such as plurality voting [44].

Splitting the activity classifier into three blocks, event processing, behavior constraints, and body elevation recognition enabled a great modularity. Each of these blocks can be tuned according to the studied pathology.

The impact of the study design on the development and evaluation of an activity-type classifier is a topic that was recursively addressed in the last years [13, 17, 45]. These studies showed that data collected in a protocol involving scripted activities under confined laboratory conditions may not reflect real-life situations. This may be particularly critical for activity-type classifiers based on machine-learning approaches (discrepancy between features extracted from ‘lab’ and ‘real-file’ data). In this study, we tried to minimize this issue by first designing a measurement protocol as similar as possible to the real-life context, i.e., self-paced various activities performed in an extended physical space (different locations in the hospital area). Second, an “expert-based” activity-classifier was designed based on biomechanical models/constraints and behavioral rules; this approach is expected to be robust in different contexts since the biomechanical/behavioral rules still stand. Although it remains to be proven, our expectation is that performances of the proposed algorithm will not change significantly with data collected from patients in home environments.

This study has however few limitations mainly due to the data available for validation. One limitation is related to the non-uniformity of the number of data samples for the different activities. The number of samples in static activities (Sit) and at Flat body elevation (i.e., more flat walking than up/down stairs) was greater due to the reduced physical capacity of patients, fatigue and fall-risk concerns (4 of 12 patients needed walking assistance). However, collected data corresponds to real-life context, both in terms of protocol design (different self-paced activities in an extended area of the hospital) and patients’ clinical condition. Another limitation of this study included the small sample size which may have led to an under-powered statistical analysis. An extension of this work could thus be to validate this approach on a greater number of stroke patients or on another patient population impaired by mobility restriction such as patients suffering from Parkinson’s disease or chronic pain.

## Conclusion

This work reported on the development of an activity monitoring system based on a single trunk-fixed multimodal sensor that includes IMU and BP, and on an algorithm to estimated basic postures/activities. The main feature of the developed algorithm is the hierarchical fuzzy inference system; it provides a versatile activity classifier where both detected event and behavior rules can be deliberately combined to improve the activity recognition. The proposed approach showed improved performances over all other state-of-the-art fuzzy logic based algorithm and epoch-based classifiers, mainly due to an improvement in the recognition of the *standing* activity. Furthermore, it was possible to accurately classify body elevation by a decision tree and a sinus-fitting algorithm. The high CCR values for activity classification and body elevation recognition, confirms our hypothesis that the system could be useful for unobtrusive monitoring and reliable assessment of daily-life activity in stroke patients.

## Figures and Tables

**Fig. 4:**
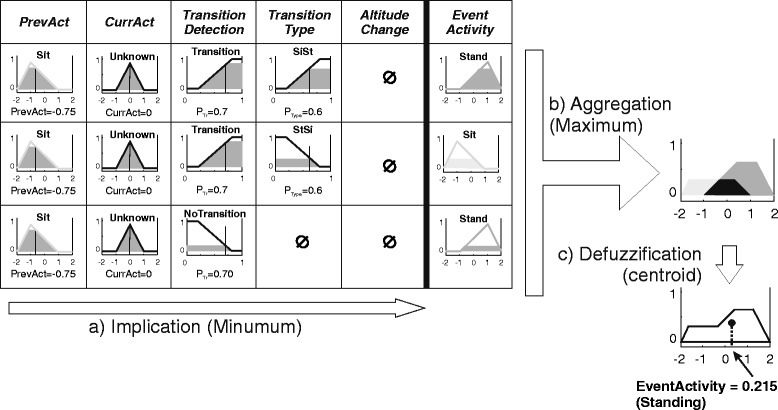
Fuzzy inference example of a Sit-to-Stand transition. First the input variables are fuzzified using the membership functions. Then for each rule, the implication function (minimum) computes the minimum degree of membership (Y-axis) across each fuzzy variable and reports into the “Event Activity” variable. The degree of membership for each rule/input is denoted by the shaded area. The “Event Activity” variables (resulting from each rule) are then aggregated by merging the shaded area (the maximum function) creating a polygon shape. The defuzzification consists of computing the centroid value of this shape

**Fig. 5:**
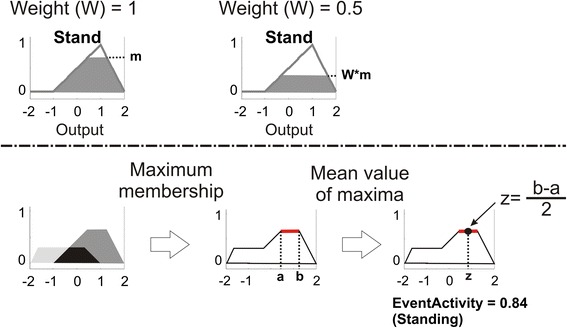
Fuzzy inference system: weighting membership function and defuzzification. (*Top*) Fuzzy output for rules with two different weights W = 1 on the top left graph and W = 0.5 on the top right axis. (*Bottom*) Defuzzification of the H-FIS: Mean of maximum. It corresponds to the middle value of the maximum membership plateau

## Appendix

### Fuzzy logics

A fuzzy inference system is generally defined by a set of membership functions to transfer its inputs into fuzzy (linguistic) variables, a set of “If-Then” rules to fuse the fuzzy variables and map the antecedents to consequences and, an implication and aggregation operator, and finally a de-fuzzification method. As an example, the first rule from Table 2 should be read as: “If the *PrevAct* is *sitting* AND *CurrAct* is *unknown* AND *Transition* is *Detected* AND *TransitionType* is *SiSt* THEN *EventActivity* is *standing*”. As another example, a fuzzy inference system (used for classification) and its fuzzy rules are presented in Fig. 4. This fuzzy inference system works as follows. The first step is to fuzzify the input using the membership functions. In this example, we assumed following values: *PrevAct* = −0.75 (fell into sit, unknown and stand activities with decreasing membership values respectively); *CurrAct* = 0 (fell into unknown, sit and stand activities); *Transition* = 0.7; *TransitionType* = 0.6. Their degrees of membership are computed through the membership function as showed in Fig. 4 for the associated rules (e.g. *TransitionType* with *SiSt*). The corresponding degree of membership for each variable is denoted by the shaded area in each graph of the input variables. They are for example: 0.88 for the *PrevAct* to be sitting, 1 for *CurrAct* to be member of Unknown, 0.83 for the *Transition* to be *Detected* and 0.66 for *TransitionType* to be *SiSt*. To compute the contributions of each variable to the rule, an implication operator is used: the minimum computed across each variable. The result of each rule is reported as a fuzzy output with a degree of membership corresponding to this minimum value. In this example and for the first rule, the minimum is 0.66 (from *TransitionType*). For the second and third rules, the fuzzy output of these rules (minimum values) were also computed: 0.33 for the 2^nd^ rule and 0.12 for the 3^rd^ rule. The next step is to bring together the contributions from each rule: the aggregation step. In our case, the maximum operator was used to merge the contributions and a polygon shape is therefore obtained as shown in Fig. 4. The last step, call the defuzzification step, computes the output of the FIS from this polygon. For the Event-FIS, the output correspond to the (x-axis value of the) centroid of polygon. Furthermore, weights were associated with the rules to change their contributions according to the confidence level of the rule. This provides an opportunity for to favor one rule with respect to another. An example of a weighted rule output is presented in Fig. 5.
